# Decreased Interfacial Dynamics Caused by the N501Y Mutation in the SARS-CoV-2 S1 Spike:ACE2 Complex

**DOI:** 10.3389/fmolb.2022.846996

**Published:** 2022-07-22

**Authors:** Wesam S. Ahmed, Angelin M. Philip, Kabir H. Biswas

**Affiliations:** ^1^ Division of Biological and Biomedical Sciences, College of Health and Life Sciences, Hamad Bin Khalifa University, Qatar Foundation, Doha, Qatar; ^2^ Division of Genomics and Translational Biomedicine, College of Health and Life Sciences, Hamad Bin Khalifa University, Qatar Foundation, Doha, Qatar

**Keywords:** ACE2, COVID-19, molecular dynamics simulation, SARS-CoV-2, S1 spike protein, N501Y mutant

## Abstract

Coronavirus Disease of 2019 (COVID-19) caused by Severe Acute Respiratory Syndrome Coronavirus 2 (SARS-CoV-2) has resulted in a massive health crisis across the globe, with some genetic variants gaining enhanced infectivity and competitive fitness, and thus significantly aggravating the global health concern. In this regard, the recent SARS-CoV-2 alpha, beta, and gamma variants (B.1.1.7, B.1.351, and P.1 lineages, respectively) are of great significance in that they contain several mutations that increase their transmission rates as evident from clinical reports. By the end of March 2021, these variants were accounting for about two-thirds of SARS-CoV-2 variants circulating worldwide. Specifically, the N501Y mutation in the S1 spike receptor binding domain (S1-RBD) of these variants have been reported to increase its affinity for ACE2, although the basis for this is not entirely clear yet. Here, we dissect the mechanism underlying the increased binding affinity of the N501Y mutant for ACE2 using molecular dynamics (MD) simulations of the available ACE2-S1-RBD complex structure (6M0J) and show a prolonged and stable interfacial interaction of the N501Y mutant S1-RBD with ACE2 compared to the wild type S1-RBD. Additionally, we find that the N501Y mutant S1-RBD displays altered dynamics that likely aids in its enhanced interaction with ACE2. By elucidating a mechanistic basis for the increased affinity of the N501Y mutant S1-RBD for ACE2, we believe that the results presented here will aid in developing therapeutic strategies against SARS-CoV-2 including designing of therapeutic agents targeting the ACE2-S1-RBD interaction.

## Introduction

Severe acute respiratory syndrome coronavirus 2 (SARS-CoV-2) is a positive-sense, single stranded, enveloped RNA virus that belongs to the Coronaviridae family and is the causative agent of the coronavirus disease 2019 (COVID-19). (Wu et al., 2020) As of October 2021, more than 245 million confirmed cases have been reported worldwide, with more than five million deaths (https://covid19.who.int/). In general, coronaviruses express four structural proteins: nucleocapsid (N) protein that encapsulates the genomic material; membrane (M) protein that promotes the membrane curvature to bind to the N protein; envelope (E) protein which ensures virus assembly and release; and envelope-anchored spike (S) glycoprotein that protrudes from the viral surface and facilitates viral attachment and entry into host cells. (V’kovski, 2021; Hussein et al., 2020; Shang et al., 2020) The latter is cleaved during viral entry into two subunits, namely S1 and S2. (Samavati and Uhal, 2020) Viral attachment to host cells occurs through binding of its receptor binding domain (RBD) - which is part of the S1 subunit – to the host cell membrane-localized angiotensin converting enzyme 2 (ACE2) receptor. It is important to note that the affinity of SARS-CoV-2 S1-RBD for ACE2 was reported to be 10 times higher than that of SARS-CoV, providing a biochemical basis for the increased infection efficiency of SARS-CoV-2 compared to SARS-CoV. (Andersen et al., 2020) In this regard, computational studies have revealed an expanded network of hydrogen bond (H-bond) and hydrophobic interactions formed at the interface of ACE2-S1-RBD complex in SARS-CoV-2. (Spinello et al., 2020; Wang et al., 2020) Given these, the ACE2-S1-RBD interaction has become an attractive target for developing inhibitors of viral entry into host cell. (Andersen et al., 2020; Choudhary et al., 2020; Shang, 2020; Walls, 2020; Wrapp et al., 2020) For instance, the human recombinant soluble ACE2 protein has been utilized for reducing SARS-CoV-2 binding to the cellular ACE2 receptor leading to reduced injury to multiple organs, including the lungs, kidneys, and heart. (Zoufaly et al., 2020) Similarly, monoclonal antibodies such as 18F3 and 7B11 have been developed to neutralize SARS-CoV-2 infection by blocking epitopes on the S1-RBD. (Tai et al., 2020)

On top of the increased affinity of SARS-CoV-2 S1-RBD to ACE2 compared to SARS-CoV, new genetic variants with increased infectivity and virulence, likely arising under increased immunological pressure in patients suffering from COVID-19 or convalescent plasma therapy (Avanzato et al., 2020; Choi et al., 2020), have further complicated our efforts towards thwarting the pandemic. One of the key examples of such variants is the S1-RBD D614G mutant that has outcompeted the Wuhan-Hu-1. (Hou et al., 2020; Plante, 2021; Volz, 2021; Zhang et al., 2020) A comparative study conducted by Hou *et al* observed that this variant is superior in infecting the epithelial cells and replicates in higher number than the ancestral virus. The structural analysis showed that the S1-RBD containing the D614G mutation is more flexible and explores the open conformation more than the wild type (WT) protein, thus, leading to an increased affinity for ACE2. (Hou et al., 2020; Yurkovetskiy et al., 2020; Mansbach, 2021) Subsequently, a new phylogenetic group of SARS-CoV-2 (lineage B.1.1.7) was identified in the COVID-19 Genomics United Kingdom Consortium dataset with greater than 50% of the cases belonging to this new cluster (alpha variant) that has an estimated 50–70% increased transmissibility, as per epidemiological and virological investigations. (Rambaut et al., 2020; Santos and Passos, 2021) Indeed, reports of the presence of this variant has emerged from other countries as well. Sequence analysis indicates the presence of a total of 17 mutations spanning the ORF1ab, spike, and the N protein in the genome of this variant. (Santos and Passos, 2021) Majority of these mutations (8 out of the total 17), however, are present in the spike protein. These include deletion mutations (∆H69, ∆V70 and ∆Y144) and missense mutations (N501Y, A570D, P681H, T716I, S982A and D1118H). Of these, the N501Y substitution strikes out as one of the most interesting mutations due to its presence at the ACE2-S1-RBD interaction interface (Lan et al., 2020), raising the possibility of an altered interaction between the two proteins. In fact, deep mutational analysis of S1-RBD (Procko, 2020; Narayanan and Procko, 2021), in combination with the yeast-surface-display platform, has revealed an increased affinity of the N501Y mutant S1-RBD to ACE2 (apparent *K*_d_ of 3.9 × 10^–11^ M for the WT vs 2.2 × 10^–11^ M for the N501Y mutant). (Starr et al., 2020) Furthermore, some computational studies suggest higher binding affinity for the N501Y S1-RBD mutant to ACE2 as a result of increased coordinated hydrophobic interactions between Y501 of S1-RBD and Y41 and K353 of ACE2. (Luan et al., 2021; Spinello et al., 2021) In addition, a recent study demonstrated the EC_50_ of the mutant S1-RBD possessing a total of nine mutations (I358F, V445K, N460K, I468T, T470M, S477N, E484K, Q498R, N501Y) was nearly 17 times lower than that of the WT S1-RBD. (Zahradník, 2021)

The emergence of the B.1.1.7 alpha lineage has coincided with two independents viral evolutions, the B.1.351 (beta) and P.1 (gamma) lineages of SARS-CoV-2, all of which share the N501Y mutation in S1-RBD. The emergence of these lineages elicited new concerns regarding the evolutionary capacity of the virus. (Liu, 2021a; Tian, 2021a; Martin et al., 2021) Since December 2021, these variants have been collectively referred to as variants of concern (VOC) by the World Health Organization (WHO). By the end of March 2021, these lineages were accounting for about two-thirds of the circulating variants worldwide (Huang et al., 2021). The currently ongoing convergent evolution of N501Y lineage has led viruses to broaden the fitness landscape. (Martin et al., 2021) Structural biological studies of the SARS-CoV-2 S1-RBD proposes that N501Y mutation may increase its affinity for ACE2 binding (Starr et al., 2020; Ostrov, 2021) and that the open conformation of the N501Y mutant spike protein (Teruel et al., 2021) is associated with more efficient viral entry, transmission and infection (Leung et al., 2021). N501Y and deletion of codons 69–70 have shown a consistent fitness advantage for replication in the upper airway in the hamster model, with higher shedding in nasal secretions, as well as in primary human airway epithelial cells. (Liu, 2021a) Additionally, S-proteins of the three N501Y S1-RBD VOCs (B.1.1.7, B.1.351, and P.1 lineages) possess increased infectivity in cells expressing mouse ACE2 (Li et al., 2021). Hence, it is conceivable that mice are susceptible to the newly emerging, high frequency N501Y mutation. (Huang et al., 2021; Justo Arevalo et al., 2021) Further, this serves as an evidence for the constantly evolving SARS-CoV-2 with more contagious mutations spreading rapidly with the possibility of increasing host range. (Liu, 2021b; Chen et al., 2021; Liu et al., 2021)

The N501Y substitution alone had a phenotype similar to that of the combined eight mutations (Δ69-70, Δ145, N501Y, A570D, P681H, T716I, S982A, D1118H), suggesting that it is the major spike determinant driving increased transmission of the United Kingdom variant. Surface plasmon resonance (SPR) experiments on immobilized WT and mutant S1-RBDs (N501Y and triple mutant N501Y, K417N, E484K) demonstrated a 10-fold increased affinity to ACE2 receptor. Further, the impact of K417N and E484K was verified by single point mutations which clearly suggested a minimal impact on ACE2 binding. These highlights the vital role of N501Y in increasing the binding affinity to ACE2, thereby decelerating rate of dissociation from the ACE2 receptor in comparison to the WT. (Tian, 2021a; Istifli et al., 2021; Villoutreix et al., 2021) Computational studies by Socher et al., showed increased contact at 501 when tyrosine is present. (Socher et al, 2021) Additional studies have shown high number of contacts formed by residues F486, Y489, T500 and Y505 with ACE2 receptor. (Wan et al., 2020) Recently, the spread of a new SARS-CoV-2 spike N501Y variant harboring a set of amino acid substitutions including L18F, L452R, N501Y, A653V, H655Y, D796Y, G1219V ± Q677H in western European countries including Turkey, Nigeria, and especially France, suggests the continuous emergence of a new 501Y lineages. (Colson, 2021)

In the current study, we performed multiple all atom, explicit solvent MD simulations to gain insights into the mechanism underlying the increased affinity of the N501Y mutant S1-RBD for ACE2. Simulations of the WT and the N501Y mutant S1-RBD in complex with ACE2 showed a prolonged and stable interaction between the Y501 residue with the neighbouring Y41 and K353 residues in ACE2 in the mutant complex as compared to the N501 residue in the WT complex. Importantly, these simulations also revealed a localized decreased dynamics for interfacial residues in the mutant as compared to the WT complex that led to changes in interfacial interactions of these residues, although these were most noticeable for residues near the N501Y S1-RBD mutation site.

## Materials and Methods

### ACE2-S1-RBD Structure Preparation

The three-dimensional structure of ACE2-S1-RBD complex spanning residues S19 to D615 of human ACE2 and T333 to G526 of SARS-CoV-2 S1 glycoprotein was obtained from the RCSB PDB database as a PDB file (PDB ID: 6M0J). (Lan et al., 2020) PyMOL (The PyMOL Molecular Graphics System, Version 2.0.0, Schrödinger, LLC; pymol. org) was used to visualize the three-dimensional structure and to generate the N501Y mutant structure using the Mutagenesis tool available in PyMOL. WT and mutant PDB structure files were exported after removing ions and solvent molecules.

### ACE2-S1-RBD Molecular Dynamics Simulations

Molecular dynamics simulations were performed using NAMD version 2.13 software (Phillips et al., 2005) and CHARMM36 force field (Best et al., 2012), as described previously (Altamash et al., 2021). The simulation system consisting of the biomolecular complex formed by the ACE2-S1-RBD was generated from the previously prepared PDB files using the QwikMD Toolkit (Ribeiro et al., 2016) available as a plugin in the Visual Molecular Dynamics (VMD) (Humphrey et al., 1996) software V1.9.3. Briefly, the proteins were solvated using TIP3P (transferable intermolecular potential with three points) (Jorgensen et al., 1983) cubic water box and charges were neutralized using 0.15 M NaCl final concentration in explicit solvent with Periodic Boundary Conditions applied. The biomolecular simulation systems consisted of ∼453,000 atoms. Energy minimization was first performed for 1,000 timesteps, followed by a thermalization step where the system was slowly heated for 0.25 ns using a temperature ramp where the temperature was raised from 60 to 310 K at 1 K increment. Temperature and pressure were then maintained at 310 K using Langevin temperature control and at 1.0 atm using Nose-Hoover Langevin piston control, respectively, and a 1 ns constrained equilibration step was then performed where protein backbone atoms where constrained using harmonic potential. Finally, two independent 100 ns runs were performed for both the WT and the N501Y mutant ACE2-S1-RBD complex. A 2 fs time step of integration was chosen for all simulation where short-range non-bonded interactions were handled at 12 Å cut-off with 10 Å switching distance, while Particle-mesh Ewald (PME) scheme was used to handle long-range electrostatic interactions at 1 Å PME grid spacing. Trajectory frames were saved every 10,000 steps.

### ACE2-S1-RBD Molecular Dynamics Simulation Trajectory Analysis

Analysis of the trajectories was performed using the available tools in the VMD software. (Humphrey et al., 1996) Independent root-mean-square deviation (RMSD) calculations of backbone Cα atoms of ACE2 and S1-RBD proteins were performed using the “RMSD trajectory Tool” in VMD. (Humphrey et al., 1996) Root-mean-square fluctuations (RMSF) measurements were performed for Cα atoms of each protein. The representative composite timestep snapshot images were prepared by saving the trajectory coordinates as PDB file format every 10 ns and then combining a total of 11 frames to form the composite images. Representative trajectory movies of the 100 ns simulations were prepared from 500 trajectory snapshots (5 snapshots/ns) generated using VMD Movie Maker Tool (Humphrey et al., 1996) and compiled using Fiji distribution of ImageJ software (Schindelin et al., 2012) at a frame rate of 60 fps.

Energy calculations were performed using “NAMD Energy” analysis tool available as part of VMD. Binding free energy changes were estimated through molecular mechanics Poisson-Boltzmann surface area (MM-PBSA) method (Kollman et al., 2000) using the CaFE 1.0 tool (Liu and Hou, 2016) and VMD (Humphrey et al., 1996). Center-of-mass distances between paired selections were determined using VMD. (Humphrey et al., 1996) Dynamic Cross-Correlation (DCC) analysis was performed using the DCC algorithm from MD-TASK software suite (Brown et al., 2017) for analyzing molecular dynamics trajectories (https://mdmtaskweb.rubi.ru.ac.za/) as well as by using Bio3D R package (Grant et al., 2006; Skjærven et al., 2014). DCC calculations were based on the position of Cα atoms obtained after aligning trajectory frames on the Cα atoms of the original complex structure. Average DCC figures were prepared using MATLAB and results were represented as heat maps that indicate the range of correlations from +1 (high correlation) to 0 (no correlation) to −1 (high anti-correlation). H-bond analysis between ACE2 and S1-RBD was performed at a cut-off distance of 3.5 Å and a cut-off A-D-H angle of 20° using the “Hydrogen Bonds” analysis extension in VMD (Brielle and Arkin, 2020; Mallik et al., 2021). Interfacial residues were determined from the available ACE2-S1-RBD complex (PDB ID: 6m0j) at a cut-off distance of 5 Å using PyMOL. Standard deviations of the inter-residue distances obtained over the course of the simulation were then normalized with their respective average distances and plotted as a ratio of N501Y mutant to WT ACE2-S1-RBD complexes.

### Data Analysis and Figure Preparation

GraphPad Prism (version nine for macOS, GraphPad Software, La Jolla California United States ; www.graphpad.com), in combination with Microsoft Excel, were used for data analysis and graph preparation. Figures were assembled using Adobe Illustrator.

## Results and Discussion

**FIGURE 1:**
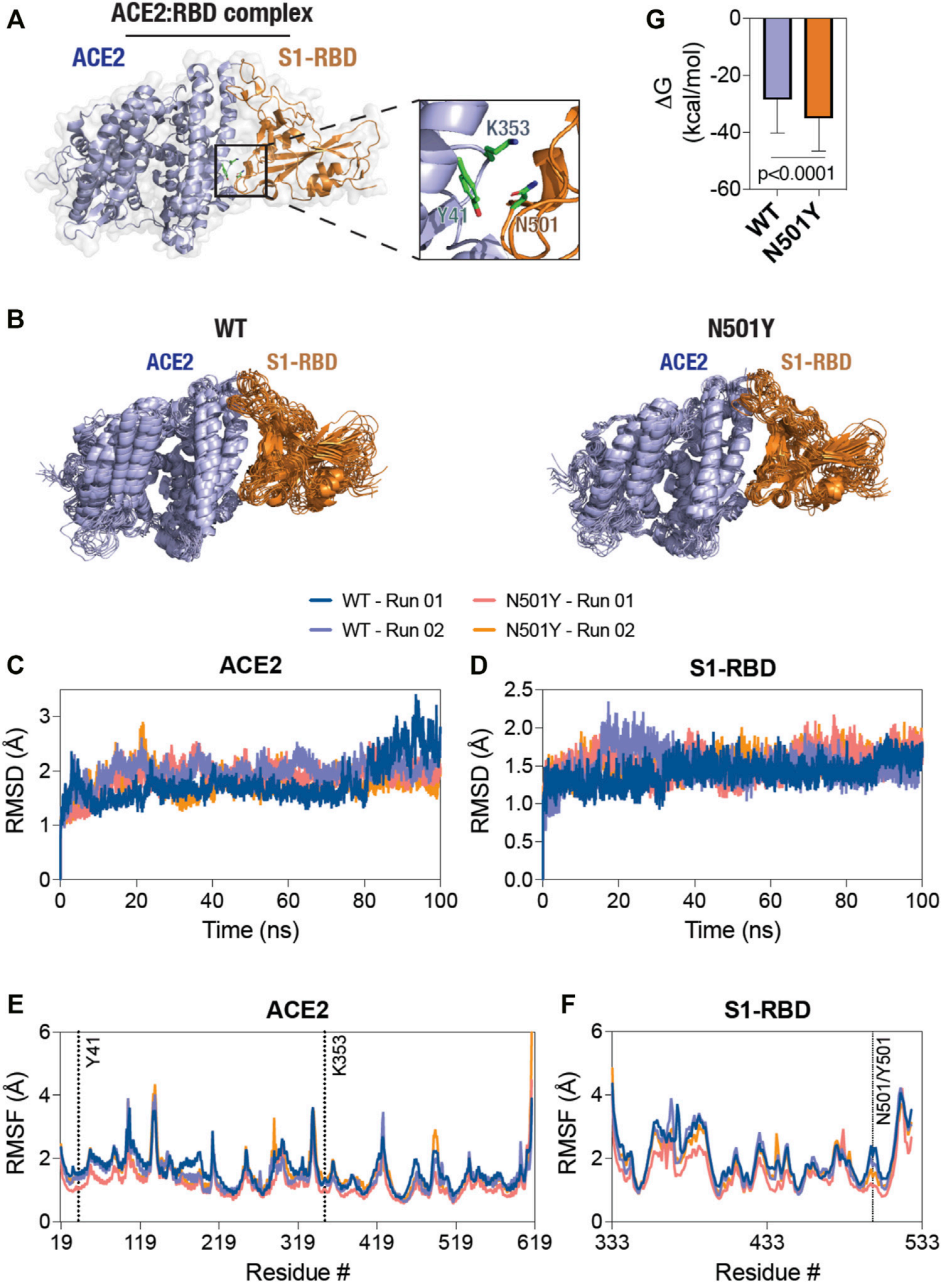
Decreased structural dynamics of the N501Y mutant S1-RBD in complex with ACE2. **(A)** Cartoon representation of the ACE2-S1-RBD structure (PDB: 6M0J (Lan et al., 2020) showing the relative positioning of residues Y41 and K353 in ACE2 (light blue) and residue N501 in S1-RBD (orange). **(B)** Cartoon representation of the WT (left panel) and the N501Y mutant (right panel) ACE2-S1-RBD complex showing structural evolution of the complex over time in a 100 ns all-atom, explicit solvent MD simulation. Composite images were prepared using 11 consecutive frames from up to 100 ns simulations with each frame being 10 ns apart. **(C, D)** Graph showing backbone (Cα) root-mean-square deviation (RMSD) values of ACE2 **(C)** and S1-RBD **(D)** obtained from the simulation of the WT and N501Y mutant ACE2-S1-RBD complexes. **(E,F)** Graph showing backbone (Cα) root-mean-square fluctuation (RMSF) values of ACE2 **(E)** and S1-RBD **(F)** obtained from up to 100 ns simulations of the WT and N501Y mutant ACE2-S1-RBD complexes. **(G)** Graph showing binding free energy changes (ΔG, kcal/mol) obtained from the last 50 ns of MD simulation using the MM-PBSA method (mean ± S.D.).

In order to understand the mechanism underlying the enhanced affinity of the N501Y mutant over the WT S1-RBD for ACE2, we initiated MD simulations with the available ACE2-S1-RBD complex structure (PDB ID: 6M0J) (Lan et al., 2020). A closer inspection of the ACE2-S1-RBD interface indicated that residues Y41 and K353 of ACE2 are in close proximity to the N501 residue of S1-RBD (Figure 1A). In fact, N501 has been reported to participate in H-bond interaction (at 3.7 Å distance) with Y41 residue of ACE2, indicating its potential role in the ACE2-S1-RBD interaction. (Lan et al., 2020) We hypothesized that this interaction at the residue-level is altered by the N501Y mutation in S1-RBD. We also hypothesized that other pair-wise interactions at the interface may be altered by the same mutation. To test these hypotheses, we initiated multiple, all-atom MD simulations in explicit solvent with the WT and the N501Y mutant ACE2-S1-RBD complex structure and analyzed the trajectories obtained for general structural dynamics and specific interactions. Further, we performed the simulations in duplicates to test the consistency of the results and for statistical support.

These MD simulations revealed a generally decreased dynamics of the N501Y mutant ACE2-S1-RBD complex compared to the WT complex as seen from the composite image of the complexes obtained from the simulation trajectories (Figure 1B). (Biswas, 2018; Biswas and Visweswariah, 2017; Biswas, 2017; Biswas et al., 2015; Fiskerstrand et al., 2012; Biswas and Visweswariah, 2011; Biswas et al., 2008) However, RMSD analysis of backbone atoms of the proteins ACE2 and S1-RBD individually, taken over the entire course of simulation, did not show any clearly discernable trend for structural evolution of amino acid residues in the complex (Figures 1C, D). This suggests that any alteration in the biochemical interaction between the two proteins likely arises due to changes in the dynamics of specific, individual residues in the proteins. Indeed, RMSF analysis of individual amino acid residues in the proteins showed several distinct changes, with a general decrease in the N501Y mutant complex (Figure 1E). Specifically, in ACE2, residue positions S106 until S128 and L176 until M190 of ACE2 showed a reduced RMSF values in the N501Y mutant complex. RMSF analysis of S1-RBD showed a reduced structural fluctuation of Y501 in the mutant complex compared to N501 in the WT complex (Figure 1F), indicating a more stable interaction with adjacent, interfacial residues in ACE2. Importantly, residue positions sequentially (Y495 until Q506) and physically (D442 until N448) adjacent to Y501 also showed reduced dynamic fluctuations, indicating a local stabilizing effect of the mutation. Additionally, residue positions from R357 until N370, F377 until T393, G404 until I434, and S459 until R466, showed reduced RMSF values in the mutant complex (Figure 1F). The latter is suggestive of the possibility of an allosteric effect of the N501Y S1-RBD mutation on the mutant ACE2-S1-RBD complex as compared the WT complex. (Biswas et al., 2008; Biswas and Visweswariah, 2011; Fiskerstrand et al., 2012; Biswas et al., 2015; Biswas, 2017; Biswas and Visweswariah, 2017; Biswas, 2018) Overall, binding free energy changes estimated using MM-PBSA method (Kollman et al., 2000) revealed higher binding energy in the mutant complex compared to the WT (Figure 1G).

**FIGURE 2:**
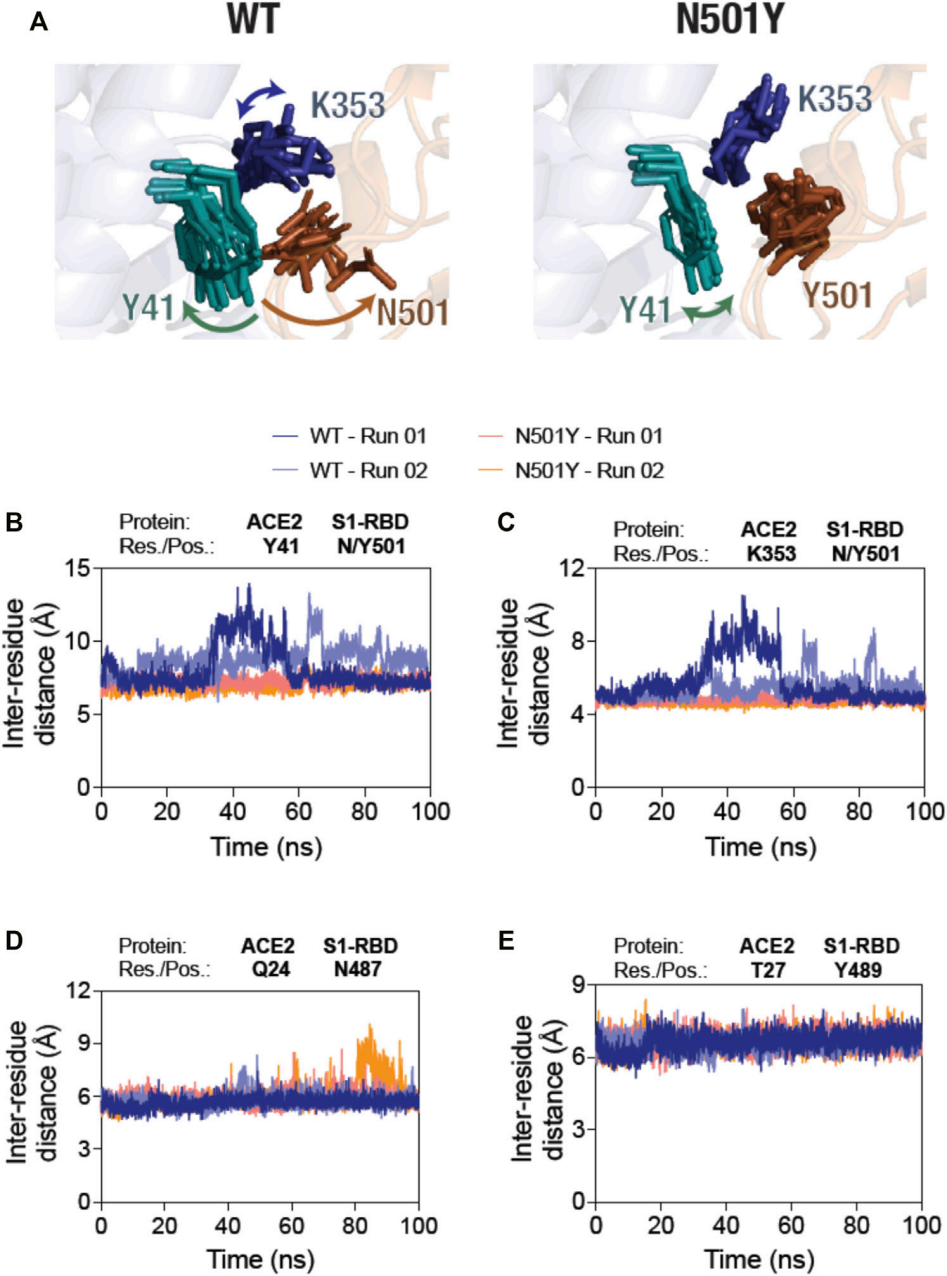
Sustained interaction of S1-RBD Y501 residue (N501Y mutant) with ACE2. **(A)** Temporal evolution of residues Y41 and K353 in ACE2 and either the N501 in the WT S1-RBD (left panel) or the Y501 in the N501Y mutant S1-RBD (right panel) in the MD simulation. A total of 11 frames obtained from up to 100 s simulations, each 10 ns apart, were compiled together. Note the increased fluctuation of the N501 residue in the WT S1-RBD. **(B–E)** Graph showing inter-residue distances between the center of masses of residue Y41 in ACE2 and N501 in the WT and Y501 in the N501Y mutant S1-RBD **(B)**, K353 in ACE2 and N501 in the WT and Y501 in the N501Y mutant S1-RBD **(C)**, Q24 in ACE2 and N487 in either the WT or the N501Y mutant S1-RBD **(D)**, and T27 in ACE2 and Y489 in either the WT or the N501Y mutant S1-RBD **(E)**. Note the increased inter-residue distance fluctuations between the residues Y41 and K353 in ACE2 and N501 in S1-RBD in the WT ACE2-S1-RBD complex compared to the N501Y mutant complex **(B,C)**.

Following these analyses, we determined the residue-residue distances based on the center-of-mass between position 501 in S1-RBD and key residues, Y41 and K353, in ACE2 of the ACE2-S1-RBD complexes, as they evolve during the span of the simulations (Figure 2A). First, N501 residue in the WT complex showed a substantially higher structural fluctuations in comparison to Y501 in the mutant complex (Figure 2A; left panel, Supporting Movies 1 and 2). This was not the case for N501Y S1-RBD mutant, in which Y501 sustained its contact at the ACE2-S1-RBD interface over the entire simulation time (Figure 2A; right panel, Supporting Movies 3 and 4). Indeed, the inter-residue distance analysis revealed a dramatic increase in the distance between Y41 and K353 in ACE2 and N501 in S1-RBD after about 30 ns in the first simulation run, while a smaller, more fluctuating, increases at different times were seen in the second run (Figures 2B, D). This is in contrast to the distances measured for the same pair of ACE2 residues with Y501 in the mutant complex (∼7 and ∼4.5 Å, respectively) (Figures 2B, D). These data suggests that Y501 residue of N501Y mutant S1-RBD forms more stable interactions at the interface with Y41 and K353 residues of ACE2 compared to the WT. To determine if the N501Y mutation impacts interaction at the opposite end of the ACE2-S1-RBD interface, we monitored the inter-residue distances between the H-bond-forming Q24 residue of ACE2 and N487 of S1-RBD and the closely juxtaposed (but not in H-bond interaction) T27 residue in ACE2 and Y489 in S1-RBD (Lan et al., 2020). In contrast to the observations made with the Y41-N501 and K353-N501 pairs, these pairs did not show substantial difference in fluctuations of their relative positioning (Figures 2D, E) compared to the mutant complex, suggesting that the effect of the N501Y mutation on the ACE2 and S1-RBD interface may be local in the timescale we have explored here.

**FIGURE 3:**
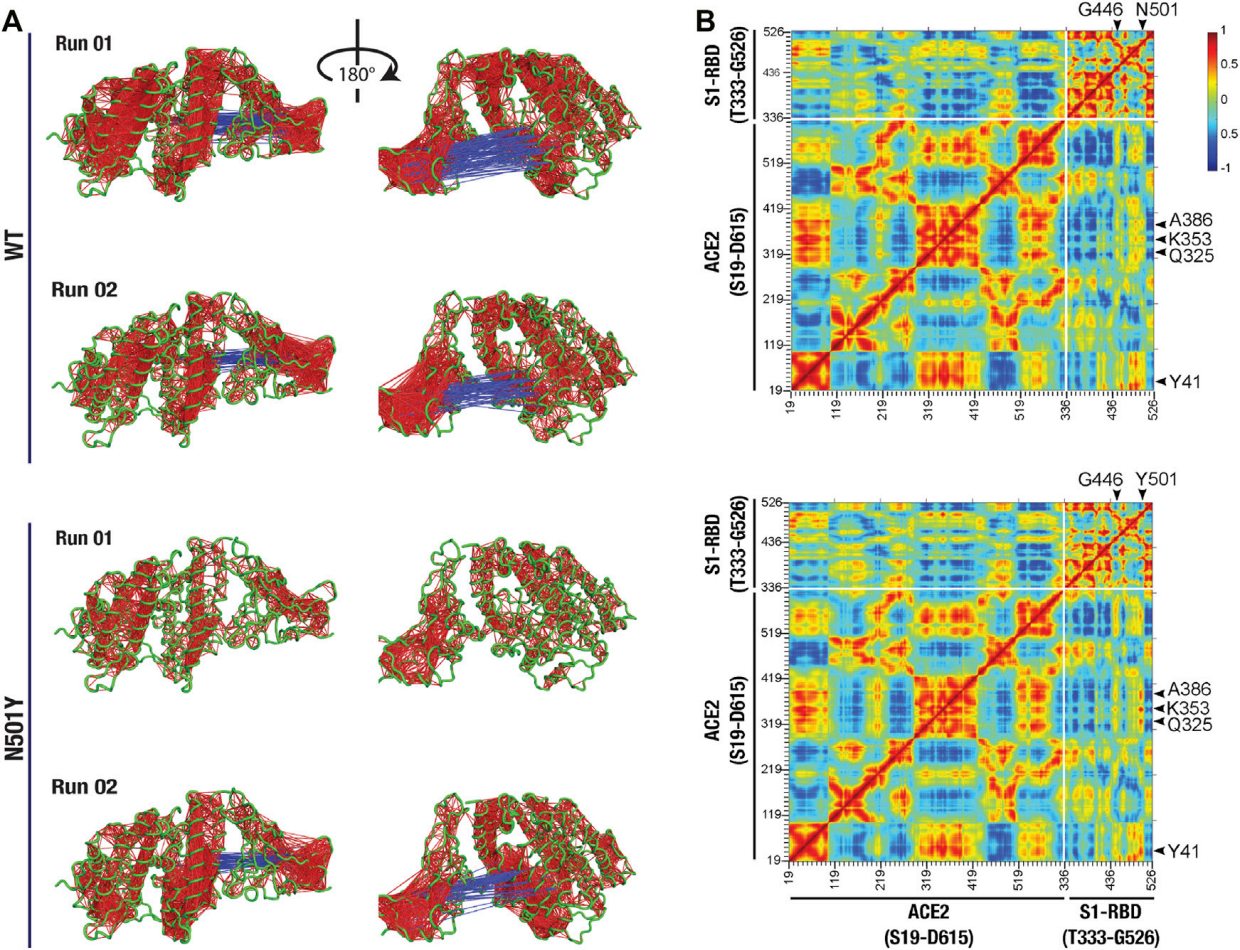
Altered dynamical cross-correlated motions in the ACE2-S1-RBD N501Y mutant complex. **(A)** Cartoon representation of ACE2-S1-RBD WT (top two panels) and N501Y mutant (bottom two panels) complex showing DCC values (cut-off, ±0.8). Note the positively correlated motions observed between Y501 and G502 on S1-RBD and residues K353 and G354 on ACE2 in the mutant complex but not in the WT complex, while less dynamically anti-correlated motions were observed in the mutant complex compared to the WT complex. **(B)** Heat map showing average DCC values from two independent 100 ns MD simulations of the WT (top panel) and the N501Y mutant (bottom panel) ACE2-S1-RBD complex (cut-off, ±0.8). Note the higher dynamically cross-correlated motions between residues at the interface in the N501Y mutant complex. Also note the global decrease in the anti-correlated motions in the mutant complex.

We then attempted to determine if there are any correlated confirmational dynamics of the complex in the WT and the N501Y mutant using dynamic cross-correlation (DCC) analysis. Application of a minimum cut-off of 0.8 to positive and negative DCC values obtained from individual MD runs showed a generally greater correlated motions (both positive as well as negative) in the WT ACE2-S1-RBD complex compared to the N501Y mutant complex. However, DCC analysis did not reveal any dynamically correlated motions between N501 of S1-RBD or any other interfacial residues located near this position and residues in ACE2 in the WT complex. Although, in the S1-RBD mutant complex, high dynamical cross-correlations were observed between residues Y501 and G502 of S1-RBD on one side and ACE2 interfacial residues, namely K353, and G354, on the other side (Figure 3A). Interestingly, application of the cut-off to the negative DCC values revealed a higher anti-correlated motions between the two chains in the WT complex compared to the mutant complex (Figure 3A). Moreover, by averaging the DCC values for the two runs, our results revealed higher dynamical cross-correlated motions between cluster of interfacial residues sequentially adjacent to the mutation site in the N501Y mutant S1-RBD (residues G496, Q498, T500, Y501, G502, V503, Y505) on one side and ACE2 interfacial clustered positions (S19, Q24, T27, F28, D30, K31, H34, E35, E37, D38, Y41, Q42, L45) (Q325, G326, N330), and (A386, R393) on the other side, compared to the WT ACE2-S1-RBD complex (Figure 3B). Similar observations were made for the DCC values between all the aforementioned ACE2 clustered positions and S1-RBD clustered residues (V445, G446, and Y449) that are physically adjacent to the mutation site as they are located on the same end of the interface as the N/Y501 clustered position mentioned earlier. Additionally, the average DCC analysis revealed a global decrease in the significantly dynamic anti-correlated motions in the mutant compared to the WT complex (Figure 3B). These results provide insight on the effect of the N501Y mutation on the dynamics of interfacial residues adjacent, either in protein sequence or in terms of physical location, to the mutation site and the distant effect of the mutation on the dynamics of non-interfacial residues manifested as a decrease in the anti-correlated inter-chain motions in the mutant complex.

**FIGURE 4:**
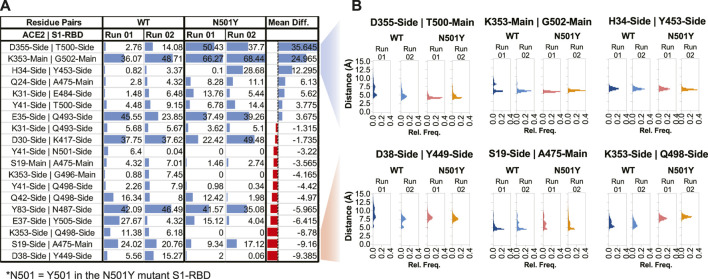
Altered H-bonding between ACE2-S1-RBD interfacial residues. **(A)** Table showing H-bonds formed at the interface by applying a cut-off of 3.5 Å distance, 20° angle, and ≥5% occupancy time. Numbers represent the % occupancy time of the H-bond during the total simulation time. **(B)** Histograms representing distance measurements between H-bond forming pairs that showed the highest alterations in H-bond formation at the interface. Mean Diff, Mean difference, i.e., difference between H-bond mean percent occupancy time of the WT and mutant complexes. Blue and red colors indicate increase and decrease in occupancy time, respectively, in the mutant complex.

In order to better understand how the two proteins interact at the interface and how this interaction compares in the WT and mutant complexes, we next performed interfacial H-bond occupancy analysis using a 3.5 Å cut-off distance and 20° cut-off angle. By applying a cut-off trajectory occupancy time of 5%, we were able to identify 19 unique H-bonds that form at the interface during the span of the simulation time by either the main chain or side chain of residues (Figure 4A). Interestingly, this analysis revealed that position 501 of S1-RBD is capable of H-bond formation with residues Y41 of ACE2 in the WT complex but not in the mutant complex. In fact, Y501 in the S1-RBD mutant complex did not form any substantial H-bonds with residues in ACE2. This indicates that Y501 residue in the mutant S1-RBD does not contribute to significant H-bond formation at the interface, but rather may be involved in forming other types of noncovalent interactions. In fact, by calculating interaction energy between this position and interfacial residues in ACE2, we found that this position forms additional, and more sustained, van der Waals interactions at the interface (Supplementary Figure S1). Recent reports suggest that this position is involved in π-π and π-cation interactions (Tian, 2021a; Ostrov, 2021). All these results are in contrast with previous reports suggesting enhanced H-bond formation by Y501 in the mutant complex (Ali et al., 2021; Tian, 2021b; Santos and Passos, 2021) driving the enhanced binding affinity of N501Y S1-RBD mutant to ACE2 (Khan, 2021; Leung et al., 2021; Zhao et al., 2021). More importantly, by calculating the difference between mean % occupancy time, we were able to determine changes in the % occupancy time between H-bonds formed in WT and mutant complexes. Interestingly, residues immediately adjacent to the 501 position in S1-RBD (T500 and G502) had the highest change in the H-bond occupancy (+35.6% and +25%, respectively), further indicating that the local effect of the mutation on the interface (Figure 4A). Distribution analysis of distances between H-bonding residue pairs that showed the highest increase and decrease in H-bond formation over the courses of the simulations revealed that the distance between these pairs generally increased and decreased, respectively, in the mutant complex (Figure 4B). More importantly, distance measurements revealed that in both cases (increased and decreased H-bond mean occupancy time) distance fluctuations between H-bond forming residue pairs decreased in the mutant complex compared to the WT complex (Supplementary Figure S2). Additionally, analysis of distance between interfacial residues that contribute to substantial H-bond interaction at the interface, but have a mean occupancy time not changing with the mutation (namely ACE2-D30-sidechain:S1-RBD-K417-sidechain, and ACE2-E35-sidechain:S1-RBD-Q493-sidechain), revealed that these residues are not located near the mutation site and display no marked differences in distance fluctuations between the WT and mutant complexes (Supplementary Figure S3), suggesting the stabilizing effect of the mutation as a key driving factor that alters H-bond interactions at the interface. The same can be concluded from calculating the distance between close-by interfacial residues at the far opposite end of the interface as was described above (Figures 2E, F).

**FIGURE 5:**
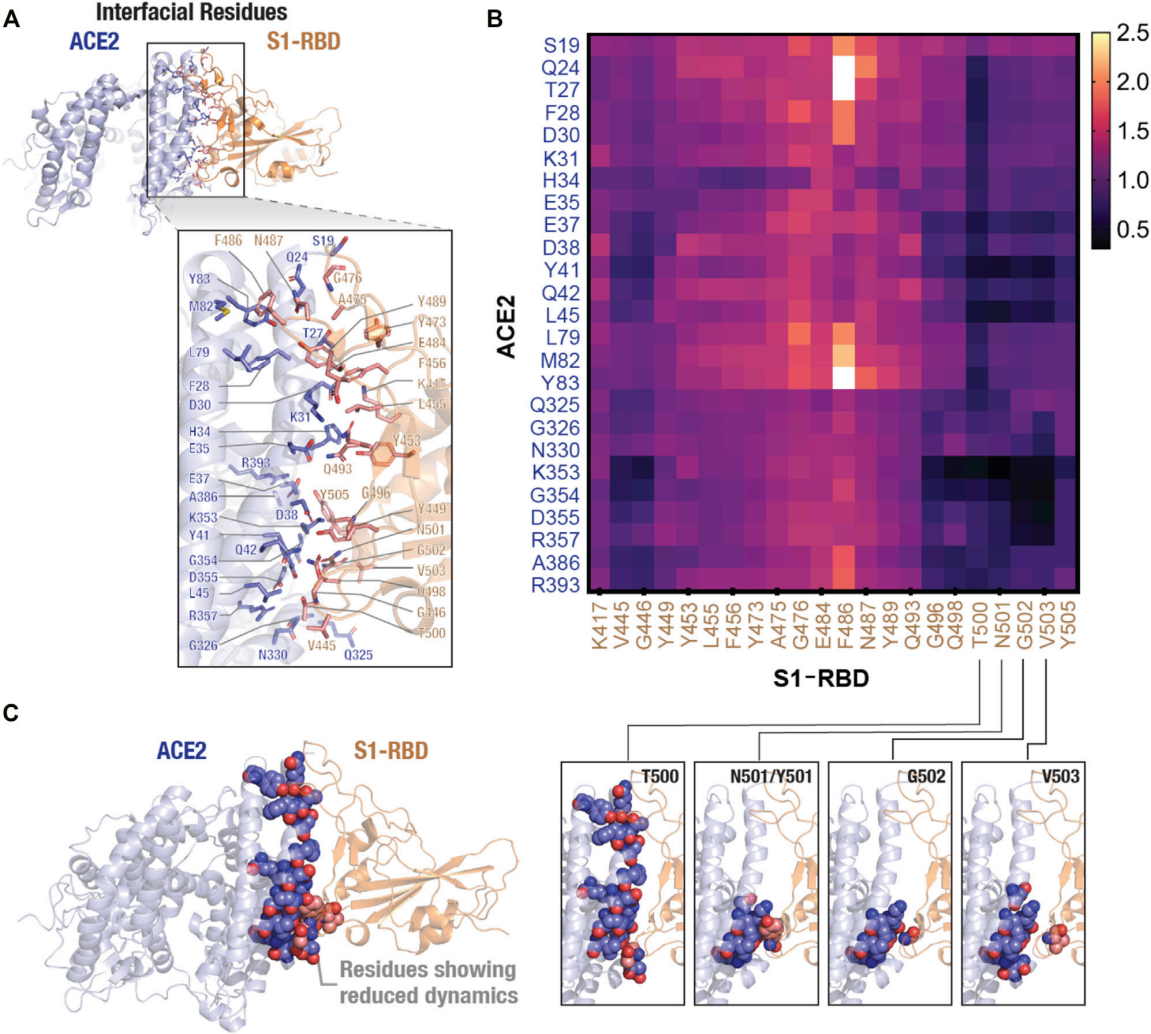
Stabilizing effect of the N501Y mutation on ACE2-S1-RBD interfacial interaction. **(A)** Cartoon representation showing interfacial residues determined from the available ACE2-S1-RBD complex (PDB ID: 6m0j) at a cut-off distance of 5Å. A total of 25 residues (blue) and 22 residues (orange) were identified at the interface in ACE2 and S1-RBD, respectively. **(B)** Heatmap representing ratio of inter-residue distance fluctuations (standard deviation normalized to average distances) in the N501Y and WT ACE2-S1-RBD complexes. Note the decrease in the dynamics of Y501 and residues neighboring the mutation site, indicating a stabilizing effect of the mutation. **(C)** Schematic showing reduced dynamics of interfacial residues in the N501Y mutant. ACE2 (blue) and S1-RBD (orange) complex showing interfacial residues with reduced dynamics in the N501Y mutant in comparison to the WT interface.

To further confirm the effect of N501Y mutation on the interface, we calculated pair-wise residue distances between residues that form the ACE2-S1-RBD interface. Using a cut-off distance of 5 Å, we were able to identify 25 interfacial residues in ACE2 and 22 in S1-RBD providing a total of 550 interfacial residue pairs (Figure 5A). The mean and standard deviation of 5,000 distance measurements (obtained from 5,000 trajectory frames) for each pair were then calculated. Standard deviations were then normalized with the mean distances for each interfacial residue pair (averaged over the two MD simulation runs) and the ratio of standard deviations obtained for the N501Y mutant and WT complexes were plotted as heatmap (Figure 5B). A value greater than 1.0 of the ratios indicate a higher pair-wise distance fluctuation, and thus, a destabilizing effect in the mutant complex compared to the WT, while a value lesser than 1.0 indicates a decreased distance fluctuation, and thus, a stabilizing effect. This analysis revealed a general stabilizing effect of the mutation on the interfacial residues with ratios ranging from 0.21 (minimum; corresponding to K353:N501 residue pairs) to 2.64 (maximum; corresponding to Q24:F486 residue pairs) with a mean of 0.86. Interestingly, the stabilizing effect was more prominent on residues that are adjacent to the mutation site either in sequence (T500, G502 and V503) or in physical proximity (V445 and G446), which further supports the idea of a stabilizing effect of the mutation on residues at the interface, including the mutated N501 residue (Figures 5B,C). Interestingly, this distance fluctuation analysis showed a maximum number of residues pairs involving T500 residue in the N501Y mutant S1-RBD, even more than residue pairs involving Y501 residue itself (Figure 5B). These results are in agreement with recent reports, both computational (Jawad et al., 2021; Socher, 2021; Villoutreix et al., 2021) as well as experimental (Liu, 2021a; Tian, 2021a; Huang et al., 2021; Li et al., 2021; Niu et al., 2021), showing an increased affinity of the N501Y mutant S1-RBD for ACE2 receptor.

After our work had become publicly available as a preprint in January 2020 (Ahmed et al., 2021), several studies reported characterization of the N501Y mutation, either alone or in combination with other SARS-CoV-2 spike mutations that exist in the VOC. For instance, Gobeil et al. (2021), using cryo-electron microscopy experiments, showed that all three VOC that contain the N501Y mutation (B.1.1.7, B.1.351, and P.1) have an increased propensity for the open-state of the spike protein, which is required for ACE2 binding, and, consequentially, an increased binding affinity for ACE2 (Gobeil, 2021). Teruel et al. (2021), using coarse-grained normal mode analysis of a large number mutants, demonstrated that the N501Y mutation alone markedly increases the SARS-CoV-2 spike open-state occupancy by increasing the flexibility of the closed-state and decreasing the flexibility of the open-state (Teruel et al., 2021) in a manner similar to that of the D614G mutation (Benton et al., 2021; Zhang et al., 2021). In fact, a computational study published in early 2020 suggested N501 residue as being compatible with, but not ideal for, human ACE2 binding (Wan et al., 2020). In addition to these, some MD simulation studies reported an enhanced binding affinity of N501Y mutant S1-RBD for ACE2 (Jawad et al., 2021; Luan et al., 2021; Spinello et al., 2021), with the possibility of a local conformational change caused by the N501Y mutation (Socher, 2021). However, such a conformational change was not observed in another MD simulation study performed with the ACE2-S1-RBD complex of the N501Y containing B.1.1.7 and B.1.531 SARS-CoV-2 variant spike protein (Villoutreix et al., 2021). In agreement with our findings, Jawad et al. (2021) (Jawad et al., 2021) showed that the N501 residue does not form substantial H-bond interaction with ACE2 residues, and that the N501Y S1-RBD mutation significantly enhances ACE2 binding by altering amino acid interactions with ACE2 at the interface. Thus, altered interfacial residue dynamics allowing for a sustained ACE2-S1-RBD interaction, likely driving the increased transmissibility of the B.1.1.7 variant, reported here appear to be consistent across multiple studies.

## Conclusion

To conclude, the MD simulations performed here with the ACE2-S1-RBD complex provide an unambiguous mechanistic insight into the increased binding affinity of the N501Y mutant S1-RBD for ACE2. Specifically, our computational work shows that the mutation of N501 residue into tyrosine (Y) results in a stable interaction with the Y41 and K353 residues in ACE2. This is positively impacted by the altered dynamics of the S1-RBD upon N501Y mutation, which is more noticeable on residues adjacent to mutation site, and extends to include certain nonadjacent residues, although the reason behind it is not entirely clear and will likely require further investigation. The N501Y S1-RBD mutation, classified as a high-frequency temporal dynamics mutation (Justo Arevalo et al., 2021), has gained tremendous interest from the scientific community given its presence in three of the SARS-CoV-2 VOC that by march accounted for more than two-thirds of the circulating variants world-wide (Huang et al., 2021). A number of studies corroborating our conclusions have appeared, which suggest the essential role of N501Y S1-RBD mutation in the transmissibility of SARS-CoV-2 variants that carry this mutation by forming a high affinity and more stable interaction at the ACE2-S1-RBD interface, possibly by altering interfacial dynamics as is evident from our study. We believe that the results outlined here will be helpful in efforts towards thwarting this new wave of COVID-19 by enabling discovery of potent inhibitors of ACE2-S1-RBD interaction (Andersen et al., 2020; Choudhary et al., 2020; Shang, 2020; Walls, 2020; Wrapp et al., 2020) or the development of high affinity ACE2 variants for use as decoys (Chan et al., 2020; Glasgow et al., 2020; Chan et al., 2021; Jing and Procko, 2021).

## Data Availability

The original contributions presented in the study are included in the article/Supplementary Material, further inquiries can be directed to the corresponding author.
