# Corrigendum: Decreased interfacial dynamics caused by the N501Y mutation in the SARS-CoV-2 S1 spike:ACE2 complex

**DOI:** 10.3389/fmolb.2022.1018464

**Published:** 2022-10-19

**Authors:** Wesam S. Ahmed, Angelin M. Philip, Kabir H. Biswas

**Affiliations:** ^1^ Division of Biological and Biomedical Sciences, College of Health and Life Sciences, Hamad Bin Khalifa University, Qatar Foundation, Doha, Qatar; ^2^ Division of Genomics and Translational Biomedicine, College of Health and Life Sciences, Hamad Bin Khalifa University, Qatar Foundation, Doha, Qatar

**Keywords:** ACE2, COVID-19, molecular dynamics simulation, SARS-CoV-2, S1 spike protein, N501Y mutant

In the published article, there was an error in **Supplementary Material**. Video files 5 to 8 with MOV file extension were updated to 1 to 4 with AVI file extension. The MOV files 5 to 8 are now removed from the **Supplementary Material**.

In the published article, there was also an error in the “**Materials and Methods**” section, sub-section “*ACE2-S1-RBD Molecular Dynamics Simulation Trajectory Analysis*”, **paragraph 1**. This sentence previously stated:

“Representative trajectory movies of the 10 ns simulations were prepared from 5,000 trajectory snapshots (50 snapshots/ns) generated using VMD Movie Maker Tool (Humphrey et al., 1996) and compiled using Fiji distribution of ImageJ software (Schindelin et al., 2012) at a frame rate of 300 fps.”

The corrected sentence appears below:

“Representative trajectory movies of the 100 ns simulations were prepared from 500 trajectory snapshots (5 snapshots/ns) generated using VMD Movie Maker Tool (Humphrey et al., 1996) and compiled using Fiji distribution of ImageJ software (Schindelin et al., 2012) at a frame rate of 60 fps.”

The authors apologize for these errors and state that this does not change the scientific conclusions of the article in any way. The original article has been updated.

