# Supplementary material for: Decreased Interfacial Dynamics Caused by the N501Y Mutation in the SARS-CoV-2 S1 Spike:ACE2 Complex
Source: Front Mol Biosci. 2022 Jul 22;9:846996. doi: 10.3389/fmolb.2022.846996 (PMC9355283; doi:10.3389/fmolb.2022.846996)
Supplement: Supplementary file 4 [file DataSheet1.docx]

## **Supporting Information**

**Decreased Interfacial Dynamics Caused by the N501Y Mutation in the SARS-CoV-2 S1 Spike:ACE2 Complex**

Wesam S Ahmed^1^, Angelin M Philip^2^, Kabir H Biswas^1,*^

**Affiliation**:

^1^Division of Biological and Biomedical Sciences, College of Health & Life Sciences, Hamad Bin Khalifa University, Qatar Foundation, Doha – 34110, Qatar

^2^Division of Genomics and Translational Biomedicine, College of Health & Life Sciences, Hamad Bin Khalifa University, Qatar Foundation, Doha – 34110, Qatar

ORCID:

Wesam S Ahmed: 0000-0002-3441-2631

Angelin M Philip: 0000-0001-7222-4669

Kabir H Biswas: 0000-0001-9194-4127

***Correspondence:** [kbiswas@hbku.edu.qa](mailto:kbiswas@hbku.edu.qa)

### **Supporting Movies**

**Supporting Movie 1.** **Trajectory movie of ACE2-S1-RBD WT complex first MD simulation run displaying interaction between ACE2 and WT S1-RBD.** Movie was created by compiling 500 snapshots over 100 ns simulation time (5 snapshots/1 ns) using a frame rate of 60 fps.

**Supporting Movie 2. Trajectory movie of ACE2-S1-RBD WT complex second MD simulation run displaying interaction between ACE2 and WT S1-RBD.** Movie was created by compiling 500 snapshots over 100 ns simulation time (5 snapshots/1 ns) using a frame rate of 60 fps.

**Supporting Movie 3. Trajectory movie of ACE2-S1-RBD mutant complex first MD simulation run displaying interaction between ACE2 and N501Y mutant S1-RBD.** Movie was created by compiling 500 snapshots over 100 ns simulation time (5 snapshots/1 ns) using a frame rate of 60 fps.

**Supporting Movie 4. Trajectory movie of ACE2-S1-RBD mutant complex second MD simulation run displaying interaction between ACE2 and N501Y mutant S1-RBD.** Movie was created by compiling 500 snapshots over 100 ns simulation time (5 snapshots/1 ns) using a frame rate of 60 fps.

### **Supporting Figures**


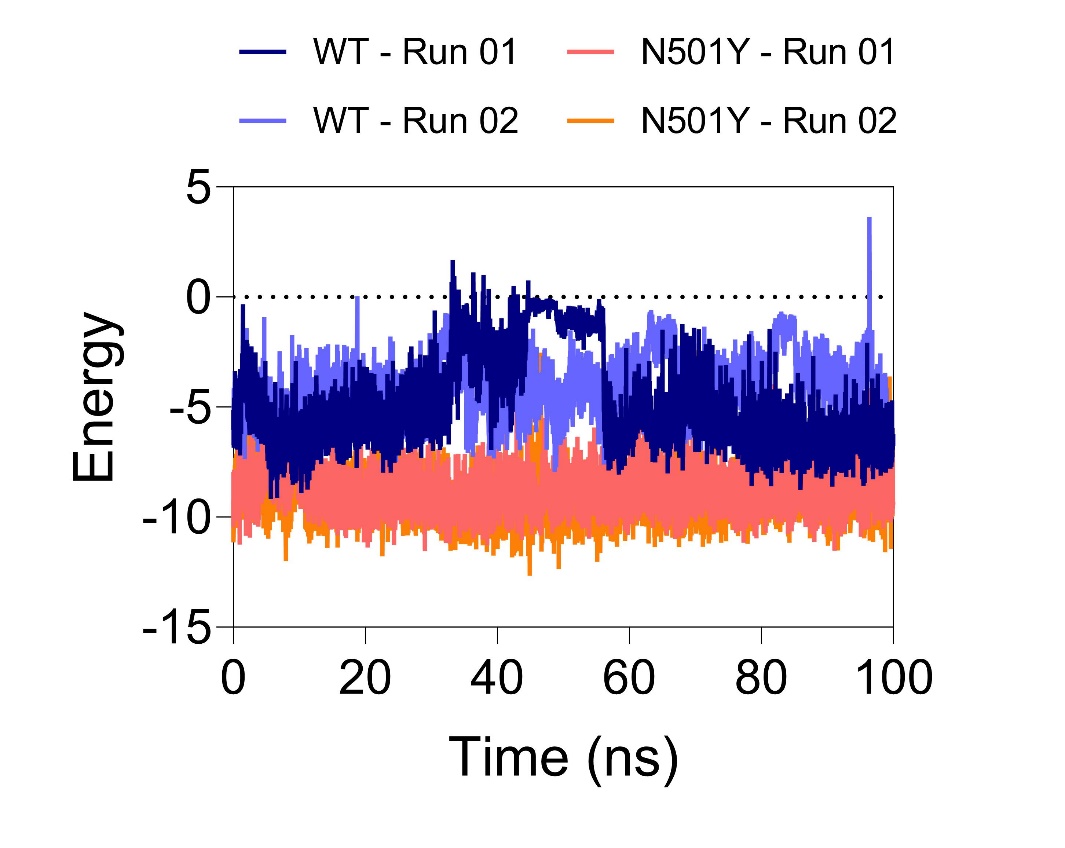


**Supporting Figure 1. van der Waals energy of interaction between position 501 of S1-RBD and residues in ACE2 in the WT and mutant complexes.**


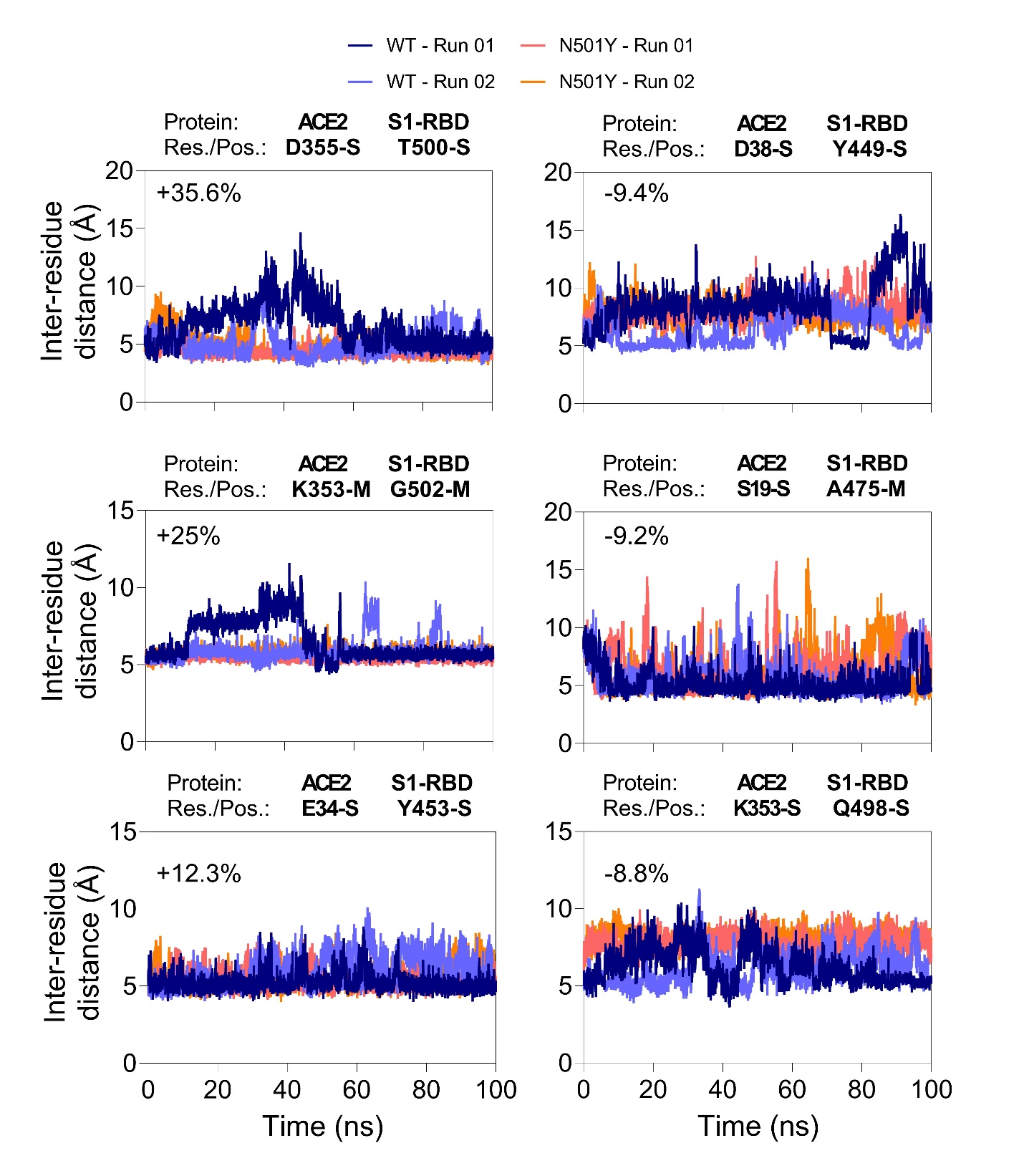


**Supporting Figure 2.** **Analysis of distance between interfacial residues whose hydrogen bond formation were most affected by the N501Y S1-RBD mutation.** On the left panel, graphs showing inter-selection (mainchain or sidechain) distances between hydrogen bond forming residues that showed the highest increase in the % mean occupancy time, arranged from the highest increase (top) to the lowest increase (bottom). On the right panel, graphs showing inter-selection (mainchain or sidechain) distances between hydrogen bond forming residues that showed the highest decrease in the % mean occupancy time, arranged from the highest decrease (top) to the lowest decrease (bottom). Note the more stable, less fluctuating, hydrogen bonds formed by pairs in the mutant complex. Inset percentages represent the increase (+) and decrease (-) in hydrogen bond mean occupancy time between the WT complex and N501Y mutant S1-RBD complexes. Res: residue, Pos: position, S: side chain, M: main chain.


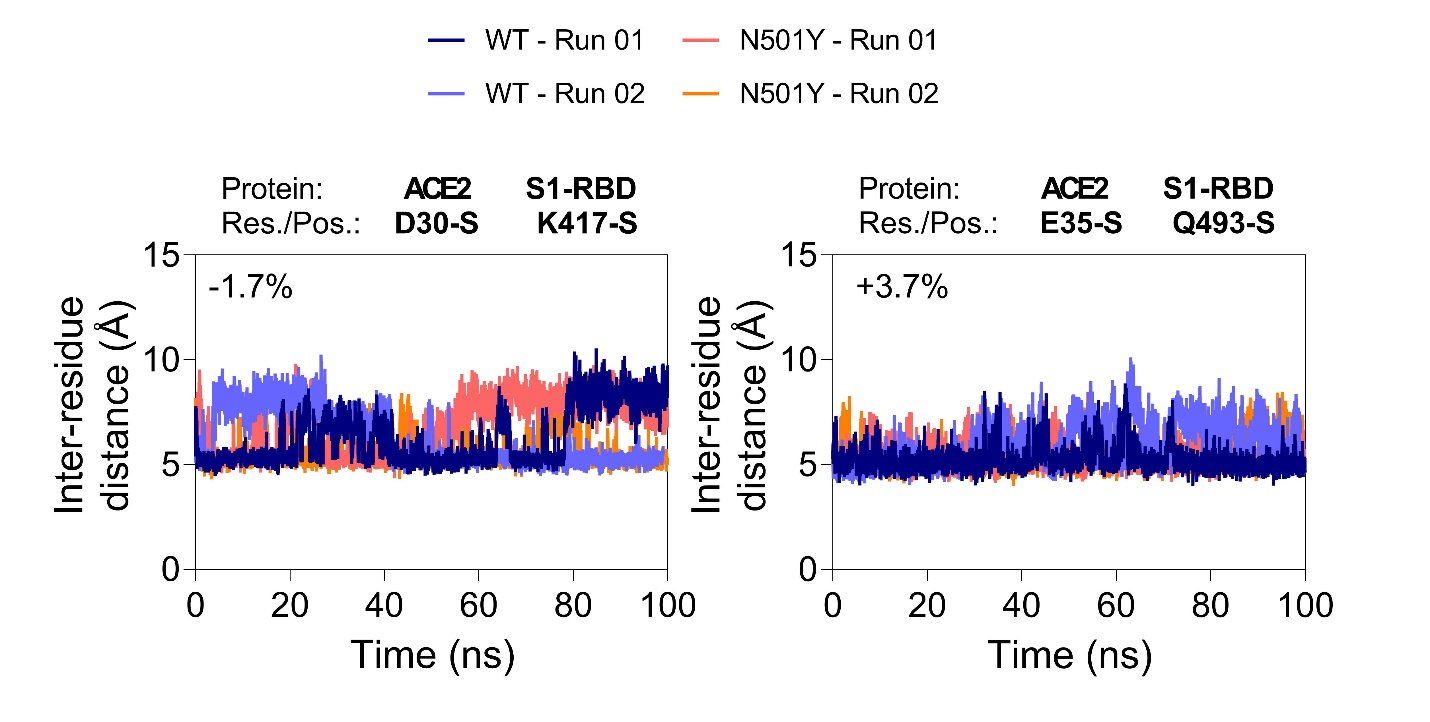


**Supporting Figure 3.** **Analysis of distances between residues that form substantial hydrogen bonding at the interface and whose hydrogen bond formation were not affected by the N501Y S1-RBD mutation.** Note the inconsiderable difference in the distance fluctuation between pairs in the WT and mutant complex. Inset percentages represent the percent change in hydrogen bond mean occupancy time between the WT and N501Y mutant S1-RBD complexes. Res: residue, Pos: position, S: side chain.
